# Extensive Deep Venous Thrombosis in a Patient with Neurolept Malignant Syndrome despite Being on Prophylaxis

**DOI:** 10.1155/2011/258172

**Published:** 2011-10-19

**Authors:** Jerrin C. Mathew, Unnikrishnan Pillai, Alexander Lacasse

**Affiliations:** Department of Internal Medicine, St. Mary's Health Center, St. Louis, MO 63117, USA

## Abstract

The risk of venous thromboembolism (VTE) in patients with Neuroleptic malignant syndrome (NMS) and those on antipsychotic medications is well established. We present here a case whereby the patient had NMS and developed extensive deep venous thrombosis (DVT) despite being on standard DVT Prophylaxis. Our case illustrates that empiric intravenous heparin for the initial few days after the onset of NMS may be considered in those with high risk of VTE, as in such patients standard DVT prophylaxis may not be sufficient. To standardize as to which patients with NMS would be at the highest risk of VTE while on standard DVT prophylaxis, the role of a standardized scoring system and a double-blind randomized trial in the future would probably be beneficial.

## 1. Introduction

Neuroleptic malignant syndrome (NMS) is a common psychiatric emergency and is classically described in patients recently started on an antipsychotic medication. We report here a case of NMS precipitated by atypical antipsychotic agent (olanzapine) associated with extensive deep venous thrombosis (DVT). Throughout his stay in the hospital, in addition to standard treatment for NMS, the patient was not physically restrained and was on DVT prophylaxis with subcutaneous heparin. On the fourth day of admission he developed venous thrombosis involving the right axillary, right subclavian, right internal jugular vein, left axillary vein, and right common femoral vein. Our case illustrates that conventional DVT prophylaxis (subcutaneous heparin) may not always be sufficient for preventing DVT in patients with NMS.

## 2. Case Presentation

A 61-year-old male with history of Alzheimer's dementia, diagnosed at the age of 50, was admitted for worsening mental status and paranoid behavior. He was started on olanzapine a few weeks prior to this admission. He was found to be lethargic, drowsy, lying down in the bed motionless. His home medications included donepezil, alprazolam, and trazodone in addition to recently started olanzapine. On physical examination he had a fever of 102.1°F, heart rate—122 beats/minute, blood pressure—106/77 mm of Hg, and respiratory rate—22 breaths/minute with 100% oxygen saturation on room air. He was drowsy lying down in bed, only opening eye to repeated verbal commands. He had opisthotonus, with diffuse rigidity; reflexes were not well elicitable in view of diffuse rigidity. The rest of system examination was noncontributory.

Laboratory data showed hemoglobin level of 12.5 mg/dL (reference range—13.5–17.5 g/dL), WBC—12.5 × 10^3^/*μ*L (reference range—4.5–10.8 × 10^3^/*μ*L), platelet—253 × 10^9^/L (reference range—150–450 K/*μ*L), and creatinine kinase (CK)—6400 units/L (reference range—38–174 units/L). Blood cultures were negative; CT scan of head, abdomen, and thorax was normal; Cerebrospinal fluid analysis was normal and the urine drug screen was negative. In view of rigidity, fever and increased CK along with the history of recently added olanzapine, a diagnosis of neuroleptic malignant syndrome (NMS) was made. He was managed in the intensive care unit and in addition to hydration was treated with intravenous dantrolene (which was later changed to oral bromocriptine). Throughout his stay in the hospital he was not physically restrained and was kept on deep venous thrombosis (DVT) prophylaxis with enoxaparin. On the fourth day of admission, the patient developed swelling of arms, neck, and right lower limb. Doppler studies revealed venous thrombosis involving the right axillary, right subclavian, right internal jugular vein, left axillary vein, and right common femoral vein. The patient was thus treated with intravenous heparin (with warfarin bridging). The standard workup for new onset DVT was negative. He had normal levels of Antithrombin III, Protein C, and Protein S. In addition he was negative for Factor V Leiden, ANA, and Lupus Anticoagulant. 

Considering the temporal sequence of events and no previous history of DVT, either NMS or olanzapine or a combination of both could be implicated as the possible etiology for the extensive venous thrombosis. At time of discharge he was back to his baseline functional state. He was discharged on warfarin for a period of six months.

## 3. Discussion

NMS is a psychiatric emergency characterized by fever rigidity, altered mental status, and rhabdomyolysis. It is reported to be associated with a mortality rate of 5–11%, which is likely due to cardiovascular instability, aspiration pneumonia, venous thromboembolism, and/or severe electrolyte imbalance [1, 2]. Both typical and atypical antipsychotic medications have been associated with NMS, and the underlying pathophysiology is related to their dopamine (D2) receptor antagonistic properties. The onset of NMS can be within hours but usually averages 4–144 days after the inciting event, which is usually either the initiation or an increase in dose of an antipsychotic medication [1, 2].

The association between conventional antipsychotics and VTE has been reported in various case repots and observational studies [3, 4]. Especially low potency antipsychotics such as chlorpromazine and thioridazine and to lesser extent high potency antipsychotics such as haloperidol have been reported to have a strong association with VTE episodes. Various studies have demonstrated strong association between clozapine and VTE also [5, 6]. Cases of DVT in association with olanzapine have also been reported [6, 7]. In addition to antipsychotics, NMS potentiates the risk of VTE. Several underlying mechanisms have been proposed to explain the association between antipsychotic drugs and VTE. Sedation induced by antipsychotic drugs can increase venous stasis and therefore probability of VTE. In addition physical restraint and catatonia during NMS can potentiate vascular damage and therefore VTE. Dehydration, fever, and rhabdomyolysis can each lead to a systemic hypercoagulable state and altered mental status [8, 9]. It has also been postulated that antipsychotic drugs may enhance platelet aggregation via either an indirect pathway involving hyperprolactinemia or secondary to an increased level of lupus anticoagulant and anticardiolipin antibodies [10]. In our patient the reason for his extensive venous thrombosis involving multiple sites, in spite of being on prophylaxis, can be due to a combination of NMS, dehydration, immobility (catatonia), and atypical antipsychotic medication olanzapine.

## 4. Summary

Therefore, in conclusion, the risk of VTE associated with NMS and atypical antipsychotic medications is well established. In a patient with NMS, in addition to the prompt discontinuation of all antipsychotic medications, whenever possible, immobility should be avoided and aggressive hydration initiated. Close monitoring for any evidence (limb swelling, pain) of venous thromboembolism should be done to ensure early detection and prompt intervention. All patients with NMS should be on standard DVT prophylaxis if not contraindicated. Furthermore, as our case illustrates, empirical intravenous heparin for the initial few days after the onset of NMS may be considered in those with high risk of VTE, as in such patients standard DVT prophylaxis may not be sufficient. To standardize as to which patients with NMS would be at the highest risk of VTE while on standard DVT prophylaxis, the role of a standardized scoring system and a double-blind randomized trial in the future would probably be beneficial.
